# Predictive Validity of Screening Tools and Role of Self-Esteem and Coping in Postpartum Depression Risk

**DOI:** 10.3390/diagnostics15091152

**Published:** 2025-04-30

**Authors:** Nadica Motofelea, Alexandru Catalin Motofelea, Ionela Florica Tamasan, Teodora Hoinoiu, Jabri Tabrizi Madalina Ioana, Maja Vilibić, Antoniu Ionescu Cringu, Brenda Cristiana Bernad, Sorin Trinc, Dan-Bogdan Navolan

**Affiliations:** 1 Doctoral School, “Victor Babes” University of Medicine and Pharmacy Timisoara, 300041 Timisoara, Romania; nadica.motofelea@umft.ro (N.M.); madalina.plic@umft.ro (J.T.M.I.); bernad.brenda@umft.ro (B.C.B.); 2 Department of Obstetrics and Gynecology, “Victor Babes” University of Medicine and Pharmacy Timisoara, Eftimie Murgu Square No. 2, 300041 Timisoara, Romania; navolan@umft.ro; 3 Department of Clinical Practical Skills, “Victor Babes” University of Medicine and Pharmacy Timisoara, 300041 Timisoara, Romania; tstoichitoiu@umft.ro; 4 Center of Molecular Research in Nephrology and Vascular Disease, “Victor Babes” University of Medicine and Pharmacy Timisoara, Eftimie Murgu Square No. 2, 300041 Timisoara, Romania; 5 Department XI: Pediatrics, “Victor Babes” University of Medicine and Pharmacy Timisoara, 300041 Timisoara, Romania; tamasan.ionela@umft.ro; 6 Center for Advanced Research in Cardiovascular Pathology and Hemostaseology, “Victor Babes” University of Medicine and Pharmacy Timisoara, 300041 Timisoara, Romania; 7 Department of Psychiatry, Sestre Milosrdnice University Hospital Center, 10000 Zagreb, Croatia; maja.vilibic@gmail.com; 8 School of Medicine, Catholic University of Croatia, 10000 Zagreb, Croatia; 9 Department of Obstetrics and Gynecology, Carol Davila University of Medicine and Pharmacy, 050474 Bucharest, Romania; antoniuginec@yahoo.com; 10 Saint Pantelimon Clinical Emergency Hospital, 020021 Bucharest, Romania; 11 Center for Neuropsychology and Behavioral Medicine, “Victor Babes” University of Medicine and Pharmacy Timisoara, 300041 Timisoara, Romania; 12 Department of Microscopic Morphology, Genetics Discipline, Center of Genomic Medicine, “Victor Babes” University of Medicine and Pharmacy Timisoara, 300041 Timisoara, Romania; sorin.trinc@umft.ro

**Keywords:** postpartum depression, screening instruments, maternal well-being, Edinburgh Postnatal Depression Scale, PHQ, Rosenberg Self-Esteem Scale

## Abstract

**Background/Objectives**: Postpartum depression (PPD) is a prevalent mental health disorder affecting women after childbirth, with significant adverse effects on both maternal and infant outcomes. Early detection and intervention are critical to improving health trajectories. **Material and Methods**: This narrative review compares the predictive validity of commonly used screening instruments for PPD, including the Edinburgh Postnatal Depression Scale (EPDS), Patient Health Questionnaire-9 (PHQ-9), and brief tools like PHQ-2 and PHQ-4. It also examines the role of self-esteem, assessed using the Rosenberg Self-Esteem Scale (RSES), and coping mechanisms, evaluated through the COPE Inventory, in moderating PPD risk. **Results**: Validation studies reveal variability in the performance of screening tools across different populations, emphasizing the need for contextual calibration. Low self-esteem and maladaptive coping strategies are consistently associated with higher PPD risk, with socioeconomic status (SES) further influencing these relationships. Interventions focusing on enhancing self-esteem and promoting adaptive coping, such as cognitive–behavioral therapy and psychoeducation, show promise in reducing PPD incidence. **Conclusions**: This review highlights gaps in existing research, particularly regarding screening during pregnancy, and calls for integrated predictive models incorporating psychosocial variables. Early, context-sensitive screening approaches are essential for effective PPD prevention and management.

## 1. Introduction

Postpartum depression (PPD) is a prevalent and often underdiagnosed mental health condition affecting women after childbirth, with reported prevalence rates ranging from approximately 7% to 30% in various studies [1,2,3,4]. If left untreated, PPD can adversely affect the mother’s well-being and the child’s emotional, mental, and intellectual development [5]. In the long term, mothers with PPD may face increased risks of chronic diseases and recurrent depressive episodes [6]. Studies have also shown that untreated PPD may lead to difficulties in bonding with the infant, potentially impairing the parent–child relationship and affecting long-term emotional development [7,8,9]. Thus, the early detection and provision of support are crucial in fostering positive health outcomes for both mothers and their children. Despite a broad consensus among health organizations regarding the need for routine screening in perinatal care, many guidelines still lack specificity on the practical details required to ensure effective implementation. For instance, while the American College of Obstetricians and Gynecologists (ACOG) states that “routine screening by physicians is important for ensuring appropriate follow-up and treatment” (2023) [10], it offers limited directives on how and when this should be carried out. In contrast, the UK’s National Institute for Health and Care Excellence (NICE) provides more granular guidelines by specifying who should screen (the primary provider), at which intervals screening should occur (upon first contact, at 4–6 weeks, and 3–4 months postpartum), and which questions to use [11]. Even with these guidelines, however, there remains considerable variability in actual clinical practice, partly due to uncertainty about which screening instruments perform best in predicting PPD across diverse populations.

Research consistently supports the EPDS as a leading instrument for perinatal depression, showing high sensitivity (around 89.6%) and acceptable specificity—especially with a cutoff score of 8 [12,13]. Nonetheless, other instruments (PHQ-2, PHQ-4, Beck Depression Inventory, and Antepartum Questionnaire) remain in use. Comparative data on these tools—particularly in pregnant populations—are limited, and it is unclear which performs best across different cultural, socioeconomic, and clinical contexts [14,15,16,17]. Some studies have started to explore the combined use of these tools, with some showing that a multi-instrument approach may enhance detection rates, particularly in high-risk populations [18,19,20]. Emerging evidence highlights the impact of psychosocial factors, particularly self-esteem and coping mechanisms, on postpartum depression (PPD). Self-esteem, an individual’s assessment of their worth, is linked to mental health outcomes during the perinatal period. Low self-esteem, measured using the Rosenberg Self-Esteem Scale (RSES), correlates with increased depressive symptoms in postpartum women [21,22,23,24,25,26]. Adaptive coping strategies, evaluated through the COPE Inventory, have been shown to mitigate psychological strain and reduce PPD risk by managing stress and emotional challenges.

The aim of this review is to evaluate the predictive validity of widely used PPD screening instruments, particularly the EPDS and PHQ-9, in pregnant and postpartum populations. Additionally, the review explores the influence of psychosocial factors—specifically self-esteem and coping strategies, as measured by the RSES and COPE Inventory—on PPD risk. Finally, it examines how socioeconomic status interacts with these variables, with the goal of identifying gaps in existing screening practices and recommending integrated, context-sensitive approaches for the early detection and prevention of postpartum depression.

## 2. Materials and Methods

### 2.1. Literature Search Strategy

A comprehensive literature search was conducted to identify studies evaluating screening instruments for postpartum depression (PPD) and their integration with psychosocial measures such as self-esteem and coping mechanisms. The search spanned electronic databases including PubMed, PsycINFO, Web of Science, Scopus, and MedLine, covering the period from January 1987 to December 2024. Keywords and Medical Subject Headings (MeSH) terms were combined using Boolean operators (AND, OR) and included terms such as “postpartum depression”, “perinatal depression”, “screening”, “Edinburgh Postnatal Depression Scale (EPDS)”, “Patient Health Questionnaire-9 (PHQ-9)”, “PHQ-2”, “PHQ-4”, “Rosenberg Self-Esteem Scale”, “COPE Inventory”, “predictive validity”, “self-esteem”, “coping mechanisms”, and “socioeconomic status”.

### 2.2. Inclusion and Exclusion Criteria

Two independent reviewers screened the titles and abstracts of all identified articles, and full-text articles were subsequently assessed for eligibility according to the defined inclusion and exclusion criteria. Discrepancies between reviewers were resolved through discussion and consensus. Data extracted from each study included study characteristics (such as author(s), publication year, country, study design, and sample size), participant demographics (including age, stage of pregnancy or postpartum period, and socioeconomic status), the specific screening instruments evaluated along with their validation metrics, details regarding the assessment of self-esteem and coping strategies, and any reported interactions between these psychosocial measures and the performance of depression screening tools. The extracted data were organized in summary tables to facilitate a comparative analysis (Table 1).

A narrative synthesis was then conducted to compare the predictive validity of the various screening tools. Data were tabulated to highlight key metrics such as sensitivity, specificity, and cutoff values across different populations and settings. In addition, the review examined how psychosocial variables, specifically self-esteem and coping strategies, moderated or mediated the performance of these screening tools and influenced postpartum depression outcomes. Due to heterogeneity in study populations, methodologies, and outcomes, a formal meta-analysis was not performed. Instead, the findings were synthesized descriptively, with the aim of informing recommendations for clinical practice and guiding future research.

### 2.3. Screening Instruments Included in the Review

#### 2.3.1. Edinburgh Postnatal Depression Scale (EPDS)

The EPDS is a 10-item self-report questionnaire specifically developed to assess depressive symptoms in postpartum women, with each item scored on a scale from 0 to 3 [27]. Its design focuses on capturing the unique emotional and psychological experiences of new mothers, distinguishing it from more general depression scales. The EPDS has been rigorously validated worldwide and translated into numerous languages, which makes it a cornerstone in both clinical practice and research. Studies have demonstrated its sensitivity to treatment effects; for example, research evaluating the efficacy of zuranolone showed statistically significant improvements in depressive symptoms as early as Day 3, with reductions sustained through Day 45 in comparison to the placebo [28]. Furthermore, the EPDS has been validated against established instruments such as the Center for Epidemiologic Studies-Depression Scale (CES-D) [29] and is adaptable to different cultural contexts—an essential feature for its use in diverse populations.

#### 2.3.2. Patient Health Questionnaire-9 (PHQ-9)

The PHQ-9 is a widely utilized instrument that consists of nine items aligned with the diagnostic criteria for major depression [30]. It offers a quantitative measure of depressive symptom severity and has demonstrated high internal consistency and reliability in large, diverse samples [31]. Evidence supports the PHQ-9’s responsiveness to clinical change, with significant improvements in scores observed at various time points, such as Days 8, 15, and 45 in treatment studies [28]. However, it is important to note that the optimal cutoff scores for the PHQ-9 may vary by population, necessitating contextual calibration to ensure its accuracy and clinical utility.

#### 2.3.3. Generalized Anxiety Disorder-7 (GAD-7)

While the GAD-7 is not a depression-specific instrument, it plays a critical role in assessing generalized anxiety symptoms, which are commonly observed alongside depression in postpartum populations. This 7-item scale provides a rapid evaluation of anxiety severity and is frequently used in conjunction with depression measures like the PHQ-9 to offer a more comprehensive picture of maternal mental health. The GAD-7’s brevity and ease of administration make it especially valuable in busy clinical settings, where prompt identification of anxiety symptoms can guide further evaluation and intervention.

#### 2.3.4. Brief Screening Tools: PHQ-2 and PHQ-4

For initial screening purposes, shorter instruments such as the PHQ-2 and PHQ-4 are sometimes employed. The PHQ-2, a two-item version derived from the PHQ-9, is useful for a quick assessment but may not detect milder forms of depression as effectively [32]. In contrast, the PHQ-4 combines the two items of the PHQ-2 with an additional two items from the GAD-2, thereby screening simultaneously for both depression and anxiety [33,34,35,36,37,38,39]. Although these brief tools can enhance screening efficiency, they must be used with an understanding of their limitations, particularly regarding sensitivity.

## 3. Results

Together, these instruments form the backbone of postpartum mental health screening. Their validation across various populations and settings underscores their importance in early identification, which is critical for timely intervention and improved maternal and child outcomes.

The comparative evidence from these validation studies illustrates the variability in performance of the EPDS (and, in one case, the PHQ-9) across different populations and settings. For instance, studies in the United States, such as the one by Beck et al., 2001 [40], reported high specificity (0.99) for the EPDS using a cutoff of ≥12 in postpartum women. In contrast, Flynn et al., 2011 [41], observed high sensitivity (0.92) but lower specificity (0.54) in postpartum women using a cutoff of ≥13. Similarly, in pregnant populations, Su et al., 2001 [21], in Taiwan and Castro et al., 2015 [42], in Brazil demonstrated good sensitivity (0.83 and 0.81, respectively) with varying cutoff scores (≥13 and ≥11) and specificities (0.89 and 0.74, respectively).

The evidence also underscores the importance of contextual calibration—optimal cutoff scores may differ based on demographic and cultural factors, as well as the stage of pregnancy or postpartum period. Moreover, the performance of the PHQ-9 in postpartum populations (as seen in Flynn et al. [41]) indicates that while it reliably detects depressive symptoms (sensitivity of 0.89), its specificity (0.64) might require adjustment for certain clinical settings.

Collectively, these findings highlight the need for standardized evaluation protocols and careful consideration of population-specific factors when deploying these screening instruments in clinical practice. Ensuring accurate identification of at-risk individuals is crucial for timely intervention, ultimately improving maternal and child health outcomes (Table 2).

The studies summarized here illustrate a wide range of PHQ-9 performance characteristics in perinatal populations, with varying sensitivities and specificities based on sample demographics and chosen cutoffs. For example, Hanusa et al., 2008 [43], found relatively low sensitivity (0.31) and specificity (0.06) among 29 postpartum women at a cutoff of ≥10, whereas Flynn et al., 2011 [41], reported much higher sensitivity (0.89) at the same cutoff in a different U.S. postpartum sample. Gawlik et al., 2013 [47], demonstrated moderate sensitivity (0.40) but high specificity (0.92) in pregnant women at a cutoff of ≥10. Green et al., 2018 [53], in Kenya, tested a higher cutoff (≥15) and found balanced but moderate sensitivity (0.70) and specificity (0.74). Meanwhile, van Heyningen et al., 2018 [54], in South Africa, observed a sensitivity of 0.80 and specificity of 0.82 at ≥10 in pregnant women, suggesting that the PHQ-9 can perform well in low- and middle-income settings with appropriate calibration.

Collectively, these findings highlight that the optimal PHQ-9 cutoff can differ by context and population. Differences in cultural norms, the timing of assessment (pregnancy vs. postpartum), and sample characteristics (age, socioeconomic status) may account for variability in diagnostic accuracy. Consequently, clinicians and researchers should consider local validation studies or pilot testing to determine the most appropriate PHQ-9 threshold for identifying perinatal depression in a given setting (Table 3).

## 4. Discussion

**Figure 1:**
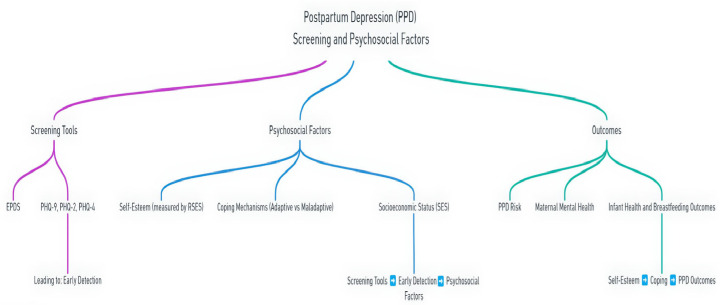
Conceptual framework of postpartum depression (PPD) screening, psychosocial factors, and health outcomes.

This study aimed to evaluate the predictive validity of postpartum depression (PPD) screening tools and to explore the critical psychosocial factors—particularly self-esteem, coping strategies, and socioeconomic influences—that affect maternal mental health outcomes. Our findings highlight the variable performance of validated screening instruments across different populations, emphasizing the need for contextual calibration. Furthermore, the results demonstrate that self-esteem and coping mechanisms are not only independently associated with postpartum depression but also interact with socioeconomic factors to shape maternal vulnerability. In the following sections, we discuss the results according to the specific objectives of the study, drawing on the existing literature to interpret their clinical and public health implications (Figure 1).

### 4.1. Predictive Validity of Screening Tools for PPD

This review highlights the crucial role of validated screening instruments such as the Edinburgh Postnatal Depression Scale (EPDS), Patient Health Questionnaire-9 (PHQ-9), PHQ-2, and PHQ-4 in the early identification of postpartum depression (PPD). The evidence demonstrates considerable variability in sensitivity and specificity across studies, reflecting differences in demographics, timing (pregnancy vs. postpartum), and cultural context. For instance, Beck et al., 2001 [46], reported high specificity (0.99) for the EPDS at a cutoff of ≥12 in U.S. postpartum women, while Flynn et al., 2011 [41], found high sensitivity (0.92) but lower specificity (0.54) at a cutoff of ≥13. Similarly, PHQ-9 performances ranged widely, from low sensitivity (0.31) and specificity (0.06) in Hanusa et al., 2008 [43], to balanced performance (sensitivity 0.80; specificity 0.82) in van Heyningen et al., 2018 [54]. These results emphasize the necessity of the contextual calibration of screening tools to ensure effective identification of at-risk individuals in diverse settings. Including brief tools such as PHQ-2 and PHQ-4 can enhance initial screening efficiency, but they must be used cautiously due to limitations in detecting milder depression.

### 4.2. Interactions Between Self-Esteem, Coping, and Socioeconomic Status

The COPE Inventory, originally developed by Carver, Scheier, and Weintraub, 1989 [55], is a comprehensive, multidimensional tool that assesses a range of coping strategies employed by individuals facing stress. It encompasses various subscales that differentiate between adaptive and maladaptive coping responses.

Adaptive coping strategies—such as active coping, planning, seeking instrumental support, and positive reframing—are considered constructive approaches that help individuals manage stress effectively. In perinatal populations, these adaptive methods have been linked to lower levels of postpartum depressive symptoms. For instance, research among postnatal mothers in Malaysia demonstrated that problem-focused coping strategies were negatively correlated with depressive symptoms [56]. Similarly, studies in Chinese populations have shown that positive coping styles, including detailed planning and active seeking of social support, are associated with reduced levels of postpartum depression [26].

In contrast, maladaptive coping strategies such as denial, behavioral disengagement, self-blame, and emotion-focused coping are generally less effective in alleviating stress and may even exacerbate negative emotional outcomes. Evidence from a study among postpartum women in Brazil indicated that avoidant strategies, such as distancing oneself from the stressor, were linked to increased depressive symptoms [25]. Likewise, research among Korean mothers found that emotion-focused coping strategies, including persistent rumination, were more prevalent in individuals experiencing postpartum depression [57].

By distinguishing between these two types of coping strategies, the COPE Inventory provides valuable insights into how different coping styles can influence mental health outcomes. This understanding is particularly important for developing targeted interventions aimed at promoting adaptive coping mechanisms, thereby potentially reducing the risk or severity of postpartum depression.

Interactions between self-esteem, coping, and socioeconomic status play a critical role in shaping the risk and severity of postpartum depression (PPD). Low self-esteem emerges as a potent contributor to maladaptive coping. Women who view themselves negatively or feel incompetent as new mothers are more likely to engage in avoidant behaviors—such as denying issues or withdrawing—and adopt self-punitive strategies like self-blame, which can maintain or worsen depressive symptoms [58,59]. In contrast, higher self-esteem facilitates the use of adaptive coping strategies, such as reaching out for help and actively problem-solving, which can act as a buffer against severe PPD [26,60]. The evidence suggests that low self-esteem and poor coping mutually reinforce one another, creating a cycle that clinical interventions must strive to break.

Different coping strategies have distinct impacts on maternal mental health. Maladaptive approaches, including avoidance, substance use, and self-blame, are frequently associated with higher levels of PPD across diverse populations; these behaviors often reflect an overwhelmed or undervalued self, leading to functional impairments in a mother’s life [59,61]. Conversely, problem-focused coping and constructive emotion-focused strategies—such as seeking social support—are linked to less severe depressive symptoms. However, depressed mothers tend to underutilize these adaptive strategies, possibly due to cognitive biases (e.g., “no one can help me” or “I don’t deserve help”) that stem from low self-worth [25]. Therapeutically, these findings underscore the importance of teaching positive coping skills in postpartum depression treatment. For example, cognitive–behavioral therapy can reduce self-blame and avoidance by helping mothers reframe negative thoughts and engage step-by-step with their problems.

Socioeconomic status (SES) further modulates coping options and outcomes. Women with low SES often face heavier burdens such as financial strain, limited social support, and greater stigma or lack of knowledge about PPD. These factors can push them toward more passive, emotion-focused coping strategies. Although endurance or informal support, such as family or faith, may sometimes provide relief, they are frequently insufficient, especially in severe cases. In higher-SES populations, women generally have greater opportunities to choose active coping methods (e.g., attending mommy-and-me psychotherapy groups or hiring a babysitter for respite), which tend to yield better outcomes. Nonetheless, even in these groups, vulnerability to maladaptive coping persists if depressive symptoms occur. Overall, while the fundamental patterns of how self-esteem and depression interact appear similar across SES groups, the extent to which adaptive coping strategies can be activated is often lower in disadvantaged populations due to external barriers [25,62].

Low SES not only predisposes women to chronic stress by limiting resources and social support but also interacts with self-esteem to elevate the risk of PPD. For example, studies have found that financial stress and a lack of social support are significant predictors of postpartum depression among low-SES women [63]. Similarly, research among Chinese women indicates that lower educational levels correlate with a higher likelihood of engaging in maladaptive coping strategies, such as avoidance, which are associated with increased levels of PPD [26].

Moreover, several studies demonstrate that self-esteem moderates the relationship between coping and PPD risk. Research indicates that women with an external locus of control—who attribute their health outcomes to chance—are at higher risk of developing PPD. An intervention study in Iran showed that reducing chance in the health locus of control while increasing internal control improved psychological health and prevented PPD [64]. In addition, cross-sectional data reveal that problem-focused coping strategies are significantly influenced by factors such as age, the gender of children, and avoidant-focused coping, with women undergoing cesarean sections being more prone to avoidant coping, which is less effective for managing PPD symptoms [64,65]. These findings suggest that promoting an internal locus of control and encouraging problem-focused coping may lead to better mental health outcomes for women at risk of or experiencing PPD. Tailoring support based on individual factors—such as age, delivery method, and family composition—may further enhance the effectiveness of coping strategies in managing postpartum depressive symptoms.

Low self-esteem, maladaptive coping, and socioeconomic stressors form a triad that can jointly exacerbate postpartum depression. Modern research underscores that interventions for PPD should be holistic, incorporating strategies to bolster self-esteem through counseling and positive feedback, teaching effective coping skills such as problem-solving and help-seeking, and reducing socioeconomic barriers by improving access to affordable mental health resources and community support. By targeting each element of this triad, healthcare providers can design more effective prevention and treatment strategies for postpartum depression, ultimately improving outcomes for mothers and their families [26,58]. Given the importance of emotional resilience, it is crucial to understand how self-esteem and coping mechanisms interact, particularly in populations facing socioeconomic challenges.

### 4.3. Neurobehavioral Underpinnings of Self-Esteem and Coping

Building on the psychosocial patterns identified earlier, emerging evidence indicates that the neurobiological basis of self-esteem and coping mechanisms is closely tied to stress regulation systems, particularly the hypothalamic–pituitary–adrenal (HPA) axis. Chronic stress—especially prevalent during the perinatal period—can dysregulate the HPA axis, leading to sustained elevations in cortisol levels and maladaptive neuroplastic changes. Longitudinal human studies have shown that higher chronic stress during pregnancy and postpartum correlates with increased hair cortisol concentrations, suggesting prolonged biological stress activation [66].

Prolonged HPA axis activation is associated with hippocampal atrophy, amygdala hyperactivity, and prefrontal cortex dysfunction—brain regions critical for self-evaluation, emotional regulation, and adaptive coping [67,68]. Recent neuroimaging research in postpartum women reveals structural changes in the hippocampus and amygdala that are linked to perinatal depression symptoms [69]. Similarly, functional MRI studies have documented disrupted connectivity between the amygdala and prefrontal circuits, with postpartum depressed women showing exaggerated emotional reactivity and impaired regulation [70].

These stress-related neuroplastic changes have profound psychological implications. Impairments in hippocampal and prefrontal function can reduce cognitive flexibility and self-regulation, making individuals more vulnerable to persistent negative self-appraisals and maladaptive coping strategies such as rumination and avoidance. For example, mothers with heightened stress responses may exhibit greater emotional reactivity and struggle to engage in effective problem-solving or support-seeking behaviors, thereby reinforcing depressive cycles [71,72].

Maladaptive coping strategies, in turn, can further impair biological stress recovery. Rumination, for instance, has been shown to prolong cortisol elevations after stress exposure, “locking” the HPA axis into a dysregulated state and preventing normal physiological recovery [71,72]. In perinatal populations, avoidant coping and emotion-focused coping have been associated with both heightened stress markers and worse depressive outcomes [73].

Thus, the interplay between chronic stress, neuroplastic brain changes, and impaired coping creates a self-perpetuating cycle that exacerbates maternal vulnerability to postpartum depression. Interventions targeting both cognitive–emotional restructuring and physiological stress regulation—such as cognitive–behavioral therapy (CBT) combined with stress reduction techniques—may offer a more integrated and effective approach to promoting maternal mental health.

Understanding these neurobehavioral pathways emphasizes the importance of the early detection of psychosocial risk factors (such as low self-esteem and maladaptive coping) and highlights the value of preventive strategies that aim not only to modify thoughts and behaviors but also to restore biological homeostasis during the perinatal period.

### 4.4. Self-Esteem in Pregnancy and Postpartum: Assessment, Risks, and Intervention Strategies

The Rosenberg Self-Esteem Scale (RSES) is a widely recognized instrument for assessing global self-esteem, defined as a person’s overall evaluation of their worth or value. Comprising ten items rated on a four-point Likert scale, the RSES captures both positive and negative dimensions of self-perception. According to Rosenberg’s theoretical framework, individuals with high self-esteem tend to perceive themselves as valuable and competent, whereas those with low self-esteem are more prone to feelings of isolation, guilt, and diminished self-worth [74,75].

Self-esteem, often termed self-respect, is a psychological concept that reflects an individual’s positive or negative attitude toward themselves [76,77]. As Rosenberg notes, individuals with high self-esteem recognize and appreciate their own worth [74]. He believes that such individuals see and respect themselves as valuable people [74]. Self-esteem is important, as shown by its special place in Maslow’s hierarchy [75]. A lack of self-esteem can lead to various issues in social relationships [78] and affect people’s interactions with others [78]. Feelings of isolation and guilt often accompany sexual dysfunction, eating disorders [79], anxiety [80], and depression [81]. Self-esteem can fluctuate throughout life [80], and pregnancy is a period when women frequently experience low self-esteem due to the physiological, anatomical, and psychological changes they undergo [23]. A lack of self-esteem is associated with poor mental and physical health in mothers, which negatively impacts infant health and the mother–infant attachment [21,22].

Numerous factors contribute to the development of self-esteem, including genetics, age, socioeconomic status, thought patterns [24], health conditions, parental influence, childhood experiences, and more [80]. Research indicates that dissatisfaction with body image among Iranian women has significantly increased in recent years [82]. Much like self-esteem, body image evolves throughout a woman’s life, encompassing phases such as menstruation, pregnancy [83,84], breastfeeding, and the postpartum period [85]. A negative body image can lead to feelings of unattractiveness, dissatisfaction in marital relationships [86], depression [87], eating disorders, and low self-esteem [88]. Postpartum depression is more prevalent among women with a negative body image [89]. Likewise, mothers unhappy with their postpartum body image tend to have a more negative attitude toward breastfeeding [90].

Self-esteem [91] and body image are among the most significant factors affecting exclusive breastfeeding. Currently, only 40% of infants worldwide are exclusively breastfed. This is concerning, as UNICEF reports that increasing exclusive breastfeeding rates could save over 80,000 infants under the age of five from death [92]. It has been noted that women who are confident and receive adequate support from family and others tend to have a positive and sustained breastfeeding experience [93]. According to the World Health Organization (WHO), initiating and continuing breastfeeding requires counseling, along with supportive and promotional programs [94].

Counseling is a process that helps enhance an individual’s attitude, behavior, and personality. Additionally, it improves communication skills, facilitates behavior change, and boosts mental health and self-esteem empowerment [93]. CBT is a counseling technique that can be employed on its own or alongside other methods to address personality disorders, mental disorders, depression, anxiety, and poor body image [95]. In this approach, the therapist does not question the client’s feelings but instead challenges the thoughts that give rise to these feelings, explaining to clients how their thoughts lead to their emotions [96]. This method instructs mothers to refrain from prejudgment and negative assessments, encouraging them to exhibit appropriate emotional responses during stressful situations such as pregnancy, breastfeeding, and the postpartum period [96]. Through a review of the literature, we concluded that CBT is likely effective in boosting self-esteem and enhancing body image. For instance, forensic research utilizing Mekereș’ Psychosocial Internalization Scale (MPIS) has demonstrated that the psychosocial impact of post-traumatic and surgical scars—manifested in diminished self-esteem and self-confidence—can adversely affect social functioning and overall well-being [97,98,99,100]. Given the established links among low self-esteem, negative body image, and adverse health outcomes, our study investigates the effects of cognitive–behavioral pregnancy counseling on self-esteem and body image (primary outcomes) and exclusive breastfeeding (secondary outcome). Given some women’s low self-esteem and impaired body image, as well as the impact of these two factors on exclusive breastfeeding, we decided to investigate the effects of cognitive–behavioral pregnancy counseling on self-esteem, body image (primary outcomes), and exclusive breastfeeding (secondary outcome).

Studies have shown that low self-esteem correlates with higher levels of postpartum depressive symptoms, suggesting that the RSES can serve as a useful predictor of postpartum depression (PPD). In one longitudinal study of pregnant women, those who reported higher self-esteem on the RSES during gestation experienced fewer depressive symptoms in the postpartum period [21,22]. Conversely, women with lower self-esteem scores were more vulnerable to stressors such as physiological and psychological changes, leading to an increased risk of PPD [23,24].

RSES stands out as a brief yet robust tool for evaluating a key psychosocial factor that can either protect against or exacerbate postpartum depression. By integrating self-esteem assessment into routine prenatal or early postpartum screening, healthcare providers can identify at-risk mothers and offer targeted interventions—such as cognitive–behavioral therapy or psychoeducation—that aim to bolster self-esteem and mitigate the onset or severity of PPD.

### 4.5. Relationship Between Coping Strategies and PPD Outcomes

The present study highlights the role of self-esteem in shaping how women cope with stress during the postpartum period and, consequently, in influencing depressive outcomes. Our findings suggest that self-esteem acts as a protective factor against stress, with higher self-esteem associated with adaptive coping strategies, such as active problem-solving and seeking social support, which help reduce stress’s negative effects. In contrast, low self-esteem appears to contribute to maladaptive coping mechanisms, including avoidance and substance use, ultimately heightening stress levels and increasing the risk of depressive symptoms. Conversely, low self-esteem appears to predispose individuals to maladaptive coping mechanisms, including avoidance and substance use, thereby exacerbating stress and increasing the risk of depressive symptoms [101,102,103].

Consistent with prior research among college students, our synthesis confirms that low self-esteem is associated with heightened perceived stress, which in turn predisposes individuals to depression [101,104]. In the context of postpartum depression (PPD), these findings are particularly salient. Women with higher self-esteem demonstrate a greater propensity to engage in adaptive coping behaviors, such as seeking social support and practicing self-care, which are linked to lower levels of depressive symptoms [105,106].

Social support emerges as a key mediating factor in this relationship. Higher social support levels correlate with increased self-esteem and reduced depressive symptoms [105,106]. This suggests that interventions designed to strengthen social networks may indirectly bolster self-esteem and promote more adaptive coping responses. Additionally, attachment styles and family support further moderate this dynamic; individuals with secure attachment and robust family support tend to maintain higher self-esteem and experience fewer depressive symptoms compared to those with insecure attachment or limited support.

Our review also highlights the buffering effect of self-esteem on the negative consequences of neuroticism and chronic stress. In studies involving older adults, higher self-esteem was associated with reduced distress in the presence of personality vulnerabilities [102]. This protective role is likely even more critical for postpartum women, who often face elevated stress levels and neuroticism during this period. Moreover, the reciprocal relationship observed between self-esteem and anxiety—where low self-esteem both predicts and is exacerbated by anxiety symptoms—underscores the potential of interventions targeting self-esteem to simultaneously alleviate both anxiety and depressive symptoms [107].

Furthermore, research indicates that fluctuations in self-esteem, alongside transient changes in loneliness and stress, can predict short-term variations in depressive states [103]. Such findings reinforce the importance of continuous monitoring and support throughout the postpartum period. Evidence from diverse populations, including university students and family caregivers, further solidifies low self-esteem as a robust predictor of depressive symptoms [108,109]. This broadens the relevance of our findings, suggesting that self-esteem is a central construct not only in PPD but across varied contexts where stress and depression are prominent.

In addition to individual psychological factors, external stressors such as caregiving burdens and socioeconomic challenges also play a significant role. Women facing substantial external pressures are more likely to exhibit lower self-esteem and, consequently, engage in maladaptive coping strategies. The triadic relationship among self-esteem, coping mechanisms, and socioeconomic status emphasizes the need for multifaceted intervention approaches that address both internal and external determinants of PPD.

Interventions aimed at enhancing self-esteem and strengthening social support networks may promote adaptive coping strategies, ultimately reducing the incidence and severity of postpartum depression. Future research should employ longitudinal designs to further elucidate the causal pathways among self-esteem, coping strategies, and PPD, and evaluate the efficacy of integrated intervention models that address both psychosocial and socioeconomic determinants of maternal mental health.

### 4.6. Clinical Implications and Future Directions

Building upon the synthesis of screening performance, psychosocial factors, and socioeconomic influences discussed above, the findings of this review underscore the need for a comprehensive and multidimensional approach to the prevention, early identification, and management of postpartum depression (PPD). Screening tools such as the EPDS and PHQ series are valuable first steps, but their effectiveness increases significantly when combined with assessments of psychosocial factors like self-esteem, coping strategies, and socioeconomic challenges.

Healthcare providers should integrate self-esteem evaluations, such as the Rosenberg Self-Esteem Scale (RSES), into routine prenatal and postpartum care to identify women at risk of adverse mental health outcomes. Early interventions, particularly those based on cognitive–behavioral therapy (CBT), may enhance self-esteem, promote positive body image, strengthen adaptive coping mechanisms, and reduce depressive symptoms.

Given the demonstrated interaction between socioeconomic status, self-esteem, and coping strategies, particular attention should be given to women from disadvantaged backgrounds. Programs that improve access to social support networks, mental health resources, and educational interventions about coping strategies can play a critical role in mitigating PPD risk.

Future research should prioritize longitudinal designs to better establish causal relationships between self-esteem, coping mechanisms, socioeconomic status, and postpartum depression outcomes. Moreover, studies investigating culturally sensitive and context-specific interventions are essential to address the diverse needs of maternal populations worldwide. Integrated models that combine psychological support with socioeconomic empowerment strategies are likely to yield the greatest improvements in maternal and infant health.

## 5. Conclusions

This review highlights the critical role of self-esteem and coping strategies as key psychosocial determinants of maternal mental health, particularly in the context of postpartum depression (PPD). Low self-esteem was consistently associated with higher vulnerability to depressive symptoms, impaired maternal–infant bonding, and maladaptive coping mechanisms. Our findings emphasize that screening tools such as the EPDS and PHQ-9 are valuable for early detection, but their predictive validity can be significantly enhanced when integrated with assessments of self-esteem and coping behaviors. A holistic, multidimensional approach to maternal mental health—one that addresses emotional resilience, adaptive coping, and socioeconomic barriers—is essential for effective PPD prevention and management. Clinical practice should prioritize the early identification of psychosocial risk factors alongside standard depression screening, offering targeted interventions such as cognitive–behavioral therapy and psychosocial support programs. This review also acknowledges certain limitations, including the narrative nature of the synthesis and variability across included studies in terms of methodology and population diversity. Future research should prioritize longitudinal designs to establish causal pathways and validate integrated screening models across culturally and socioeconomically diverse populations. Ultimately, improving maternal mental health requires not only clinical vigilance but also proactive psychosocial empowerment, ensuring that prevention, early intervention, and support strategies are tailored to the complex realities faced by new mothers worldwide.

## Figures and Tables

**Table 1:** Inclusion and exclusion criteria.

Inclusion Criteria	Exclusion Criteria
Studies involving pregnant or postpartum women	Studies focusing solely on treatment outcomes without validation data
Use of validated depression screening tools (EPDS, PHQ-9, PHQ-2, PHQ-4)	Case reports, editorials, conference abstracts, or non-peer-reviewed sources
Reporting validation metrics (sensitivity, specificity, cutoff scores)	Studies lacking sufficient quantitative data
Incorporation of psychosocial assessments (RSES, COPE Inventory)	Articles not available in English or as full texts

**Table 2:** EDPS validations studies.

Year	Authors	Location	Subjects	Age (Mean ± SD)	Weeks Pregnant/Postpartum	Total (*n*)	Scales	Cutoff	Sensitivity (95% CI)	Specificity (95% CI)
2008	Hanusa et al. [43]	USA	Postpartum women	30.1 ± 5.8	6–8	29	EPDS	≥10	0.62 (0.36–0.82)	0.88 (0.64–0.97)
2008	Hanusa et al. [43]	USA	Postpartum women	30.1 ± 5.8	6–8	29	PHQ-9	≥10	0.31 (0.13–0.58)	0.06 (0.01–0.28)
2001	White [44]	New Zealand	Postpartum women	33.0 ± 4.6	7.9 ± 5.1 (2–29)	60	EPDS	≥9	0.70 (0.48–0.85)	0.92 (0.80–0.97)
2001	Su et al. [45]	Taiwan	Pregnant women	32.0 ± 4.0	2–3 trimester (2nd 49.7%)	185	EPDS	≥13	0.83 (0.63–0.93)	0.89 (0.83–0.93)
2001	Beck et al. [46]	USA	Postpartum women	31.0 ± 4.8	2–12	150	EPDS	≥12	0.78 (0.64–0.88)	0.99 (0.95–1.00)
2013	Gawlik et al. [47]	Germany	Pregnant women	32.8 ± 4.6	31.8 ± 4.2	273	EPDS	≥12	0.80 (0.38–0.96)	0.88 (0.84–0.91)
2012	Tandon et al. [40]	USA	Pregnant and postpartum women	24.4 ± 5.8	31.0 ± 7.3/8.2 ± 3.1	95	EPDS	≥13	0.81 (0.63–0.92)	0.98 (0.88–0.98)
2011	Fernandes et al. [48]	UK	Pregnant women	21.5 ± 2.6	35.0 ± 3.0	194	EPDS	≥13	1.00 (0.88–1.00)	0.85 (0.79–0.90)
2011	Flynn et al. [41]	USA	Pregnant women	30.0 ± 8.7	21.0 ± 9.0	81	EPDS	≥13	0.79 (0.67–0.88)	0.74 (0.54–0.87)
2011	Flynn et al. [41]	USA	Postpartum women	31.0 ± 6.0	12.0 ± 11.0	81	EPDS	≥13	0.92 (0.84–0.96)	0.54 (0.36–0.70)
2010	Tesfaye et al. [49]	UK	Postpartum women	25.3 ± 5.1	10–14	100	EPDS	≥7/8	0.82 (0.52–0.95)	0.78 (0.68–0.85)
2019	Matthey et al. [50]	Australia	Pregnant women	28.4 ± 5.0	14.2 ± 3.8	252	EPDS	≥10	0.67 (0.50–0.80)	0.97 (0.94–0.99)
2019	Naja et al. [51]	Qatar	Pregnant women	28.8 ± 5.0	1–3 trimester (2nd 48.4%)	128	EPDS	≥13	0.86 (0.71–0.94)	0.90 (0.83–0.95)
2018	Chorwe-Sungani et al. [52]	South Africa	Pregnant women	25.8 ± 5.2	27.7 ± 7.9	97	EPDS	≥10	0.68 (0.48–0.83)	0.88 (0.78–0.93)
2018	Green et al. [53]	Kenya	Pregnant and postpartum women	27.1 ± 5.9	2–3 trimester (4–24)	193	EPDS	≥16	0.70 (0.40–0.89)	0.72 (0.65–0.78)
2018	van Heyningen et al. [54]	South Africa	Pregnant women	26.8 ± 5.9	1–3 trimester (2nd 46.5%)	376	EPDS	≥14	0.86 (0.76–0.92)	0.81 (0.76–0.85)
2015	Castro et al. [42]	Brazil	Pregnant women	20–39 (80%)	2nd trimester	245	EPDS	≥11	0.81 (0.67–0.90)	0.74 (0.67–0.79)

**Table 3:** PHQ validation studies.

Year	Authors	Location	Subjects	Age (Mean ± SD)	Weeks Pregnant/Postpartum	Gold Standard	Blind	Total (*n*)	Scales	Cutoff	Sensitivity (95% CI)	Specificity (95% CI)
2008	Hanusa et al. [43]	USA	Postpartum women	30.1 ± 5.8	6–8	SCID	Unknown	29	PHQ-9	≥10	0.31 (0.13–0.58)	0.06 (0.01–0.28)
2013	Gawlik et al. [47]	Germany	Pregnant women	32.8 ± 4.6	31.8 ± 4.2	SCID	Unknown	273	PHQ-9	≥10	0.40 (0.12–0.77)	0.92 (0.88–0.95)
2011	Flynn et al. [41]	USA	Postpartum women	31.0 ± 6.0	12.0 ± 11.0	DSM-IV	Unknown	81	PHQ-9	≥10	0.89 (0.81–0.95)	0.64 (0.46–0.79)
2018	Green et al. [53]	Kenya	Pregnant and postpartum women	27.1 ± 5.9	2–3 trimester (4–24)	DSM-5	Yes	193	PHQ-9	≥15	0.70 (0.40–0.89)	0.74 (0.67–0.80)
2018	van Heyningen et al. [54]	South Africa	Pregnant women	26.8 ± 5.9	1–3 trimester (2nd 46.5%)	MINI+	Unknown	376	PHQ-9	≥10	0.80 (0.70–0.87)	0.82 (0.77–0.86)

## Data Availability

Not applicable.
